# Editorial

**DOI:** 10.1080/17590914.2024.2386884

**Published:** 2024-09-09

**Authors:** Douglas L. Feinstein

**Affiliations:** University of Illinois, USA

After a 6 month hiatus, the website for *ASN NEURO*, the official journal of the American Society for Neurochemistry, is back on line and ready to receive new submissions that will be processed with our usual rapid turn-around times. During these months, we completed negotiations and design of the website with our new publisher, Taylor & Francis, and are excited to be embarking on this new stage of the journal’s growth.

Starting our own journal was an idea discussed for many years by ASN officers and council members, which came to fruition in 2008 when we began serious efforts to identify a publisher and establish an on-line journal. The first volume was published in 2009, under the leadership of Dr Anthony Campagnoni, who remained Editor-in-Chief for 5 years, replaced by Dr Monica Carson in 2014, and myself in 2018. Both Tony and Monica valued the importance of helping authors improve manuscripts to bring them to acceptance rather than rejecting them; and to assist younger investigators whenever possible; principles I strive to maintain.

Our new publisher, Taylor & Francis holds similar values, is committed to excellence in research, and the dissemination of high quality reports throughout the world. ASN joins many other esteemed societies that are published by Taylor & Francis.

We welcome your contributions, whether Research articles, Reviews, or shorter submissions on the new developments and discoveries taking place in Neurochemistry throughout the world. *ASN NEURO* now also welcomes Method articles and Data Notes. Articles may be submitted in any scholarly format, utilising Taylor & Francis format-free submission where our journal style is applied post-acceptance.

I speak both for myself as well as the ASN officers who helped bring us to this point in saying that we are proud to be a part of the Francis & Taylor portfolio and look forward to a long and successful partnership.


Douglas L. Feinstein *University of Illinois, IL, USA* *Editor in Chief* dlfeins@uic.edu


